# Spatiotemporal distribution and influencing factors of enteric fever in China: a cluster analysis based on data from 2001 to 2020

**DOI:** 10.3389/fpubh.2025.1550904

**Published:** 2025-04-09

**Authors:** Shumei Huang, Yao Tian, Meiying Yan, Chen-Long Lv, Li-Qun Fang, Biao Kan

**Affiliations:** ^1^State Key Laboratory of Infectious Disease Prevention and Control, National Institute for Communicable Disease Control and Prevention, Chinese Center for Disease Control and Prevention, Beijing, China; ^2^State Key Laboratory of Pathogen and Biosecurity, Beijing Institute of Microbiology and Epidemiology, Beijing, China

**Keywords:** China, enteric fever, geographic distribution, clustering areas, influencing factors

## Abstract

**Background:**

Enteric fever primarily affects the southwestern and central regions of China. Although the overall incidence rate has declined, certain areas have seen an increase in cases, necessitating further investigation into their geographic distribution, clustering areas, and potential influencing factors.

**Methods:**

City-level data from 2001 to 2020 were analyzed. Spatial clustering was identified, and wavelet transform analysis explored periodic and seasonal characteristics. Determinants were identified using generalized estimating equation and distributed lag non-linear model.

**Results:**

Incidence declined from 2001 to 2008 but leveled off since 2009, shifting eastward. Two clustering areas were identified: Guangxi-Guizhou-Yunnan and Zhejiang. In the Zhejiang, incidence was negatively correlated with GDP per capita and popularization rate of safe drinking water in rural areas. Temperature and relative humidity had delayed effects on incidence in Zhejiang, showing linear or parabolic patterns. In the Guangxi-Guizhou-Yunnan, incidence was positively correlated with the proportion of water bodies. Temperature and relative humidity had delayed effects on incidence in Guangxi-Guizhou-Yunnan, and these effects exhibited fluctuating patterns.

**Conclusions:**

Over the past 20 years, enteric fever incidence in China has shown a rapid early decline but has stabilized more recently. The factors influencing enteric fever prevalence vary between clustering areas, indicating the need for region-specific measures.

## 1 Background

Typhoid and paratyphoid fever, also known as enteric fever, are classified as one of Class B notifiable infectious diseases in China. Enteric fever remains a significant important global public health problem (1), with more than a quarter of the population in many low- and middle-income countries (LMICs) at high risk of enteric fever infection (2–5). Without timely intervention, the case–fatality rate of enteric fever remains high in some underdeveloped countries (6).

Human epidemics of enteric fever occur annually in China and have been reported primarily in highly endemic regions such as Yunnan, Guizhou, and Guangxi Provinces in southwestern China, Hunan Province in central China (7, 8), and Xinjiang Uygur Zizhiqu in northwestern China (9, 10). Although the incidence rate of enteric fever in China has been declining since the 1990s, with occasional small-scale rebounds (11), an increase in the number of cases has been observed in some non-priority monitored provinces, and even gradually become new epidemic hotspot areas (12).

Previous studies have explored the main drivers of spatial and temporal patterns of enteric fever, revealing strong correlations between enteric fever risk and suitable climatic factors, which may affect bacterial contamination of food and water and lead to outbreaks of enteric fever (13, 14). Recent studies have also intensely related the effects of socioeconomic factors, such as the level of economic development, sanitation facilities, and living conditions (15, 16). The epidemiological characteristics and influencing factors of enteric fever vary between different regions (17, 18), such as the southeastern coastal regions and the western inland regions (19–22). In southeastern coastal regions, the primary infected population is migrant workers, whereas in inland provinces, local residents are the main victims of enteric fever. The primary influencing factors in the western inland regions are unsafe drinking water and consuming raw vegetables (23), whereas in the southeastern coastal regions, the main influencing factors include unsafe dietary habits such as consuming raw or undercooked seafood (24). However, systematic studies on specific influencing factors in these regions are still limited.

Current research predominantly focuses on enteric fever analysis at provincial or municipal levels, with a notable absence of nationwide studies systematically evaluating the combined effects of climatic, socioeconomic, and sanitary factors on enteric fever. Future investigations should establish differentiated clustering areas and employ multidimensional data integration to elucidate the impacts of these factors, thereby informing targeted prevention strategies. In the present study, utilizing a national database of reported human cases of enteric fever, we explored the geographic distribution, clustering areas, and periodicity of enteric fever. By using a distributed lag non-linear model (DLNM) and generalized estimating equation (GEE), we further identified the determinants associated with the geographical heterogeneity of enteric fever, which are crucial for mitigating risk and guiding the surveillance and prevention of enteric fever.

## 2 Methods

### 2.1 Data collection and management

The study data were derived from the China Information System for Disease Control and Prevention (CISDCP), encompassing laboratory-confirmed and clinically diagnosed enteric fever (typhoid and paratyphoid fevers) cases. Through standardized data leansing protocols, duplicate entries (0.45%), reporting inaccuracies (0.55%), and misdiagnosed cases (e.g., pneumonia misclassification, 0.88%) were systematically removed, resulting in exclusion of 4,948 non-compliant records. The collected data encompassed monthly and annual case reports from 2001 to 2020, as well as weekly case reports from 2005 to 2020. The diagnosis of enteric fever was based on the isolation and culture of Salmonella from blood, bone marrow, or other fluid samples or the detection of specific antibodies or a fourfold or greater increase in antibody titers.

Data on three socioeconomic factors, three meteorological factors, 19 bioclimatic factors, two hygienic factors and three land cover factors that were potentially associated with enteric fever were collected and processed at the city level (Supplementary Table 1). Among them, city-level socioeconomic data and hygienic data from 2001–2020 were provided by the National Bureau of Statistics and local statistical bureaus. The raster-type maps of daily meteorological indicators with a spatial resolution of 1 kilometer were extrapolated with the inverse distance weighted interpolation technique using approximately 395 sites across China, which were obtained from the National Oceanic and Atmospheric Administration of the United States (https://www.ncei.noaa.gov/). Annual bioclimatic data with a spatial resolution of 1 kilometer were collected from WorldClim (https://www.worldclim.org/). Three categories of land cover with a spatial resolution of 0.3 kilometers were provided by the European Space Agency (https://www.esa.int/) (25). All these raster-type maps overlapped with the vector digital maps at the city level in China, and the related covariates of each city included in our study were computed through the zonal statistical calculation technique, which was performed using ArcGIS 10.7 (Environmental Systems Research Institute Inc., Redlands, CA, USA).

### 2.2 Analysis of spatiotemporal characteristics for enteric fever

We used geographical distribution maps to display the distribution and clustering characteristics of the enteric fever epidemic. To evaluate the geographical center and temporal variations of the incidence, centroid transfer analysis was employed. Line and bar graphs were used to show the trends of cases and incidence rates over time, while bubble charts revealed the temporal dynamics of deaths caused by enteric fever. The global Moran's index was applied to quantify spatial autocorrelation at a significance level of 0.05. Spatial scan analysis using SaTScan 9.4.1 (M Kulldorff, Information Management Services Inc., Cambridge, Massachusetts) identified spatial clustering areas, which were the basis for further analyses. Line plots illustrated the incidence rate and sequential incidence rate within these clusters. Stream graphs and rose charts depicted variations in case numbers and incidence rates across different age groups (0–4, 5–17, 18–39, 40–59, and ≥60 years). Wavelet transform analysis, conducted with MATLAB R2020b (MathWorks, Natick, Massachusetts), explored the periodic and seasonal characteristics of the epidemic.

### 2.3 Modeling analysis for influencing factors associated with enteric fever

A GEE was applied to assess all the potential influencing factors associated with the annual city-level incidence rate of enteric fever. The candidate explanatory variables used in this model included three socioeconomic factors, 19 bioclimatic factors, two hygiene factors, and three land cover factors per capita. Variables with *P* values < 0.05 and variance inflation factors (VIFs) < 10 in the univariate GEE modeling analysis were included in the final multivariate analysis models. Cross-validation was conducted to verify the robustness of the final multivariate GEE model, and forest plots were generated to present the relative risk (*RR*) and its 95% confidence interval (*CI*) of each factor influencing the incidence of enteric fever. Models were constructed using R software (version 4.1; R Foundation for Statistical Computing, Vienna, Austria) with the “geeglm” package, where the Poisson distribution was used as the link function. The forest plots of the final model were implemented using the R packages “geepackage” and “forestplot,” respectively.

### 2.4 Modeling analysis for lag effects of meteorological factors on enteric fever

The lag effect of meteorological factors on enteric fever incidence was assessed with a quasi-Poisson distribution with the DLNM, which was performed in the R package “dlnm” (26). Weekly values of three meteorological factors were calculated for each city within the clustering areas. Given the significant spatial heterogeneity due to the varied exposure scope of the meteorological indicators across different cities, the raw values of temperature (or rainfall, relative humidity) were employed for each city as the input of the city-specific DLNM, and internal knots at the same percentiles in the spline function were adopted for all cities within each clustering area. This approach allowed for the acquisition of percentile-based parameter estimates, which in fact corresponded to different absolute values of temperature (or rainfall or relative humidity). A multivariate meta-analysis using the restricted maximum likelihood method was subsequently conducted to integrate the city-specific estimation of clustering areas for each meteorological factor derived from the DLNM (27, 28), which was implemented in the R package “mvmeta.”

## 3 Results

### 3.1 Epidemic dynamics of enteric fever in the last 20 years and geographical clustering

**Figure 1:**
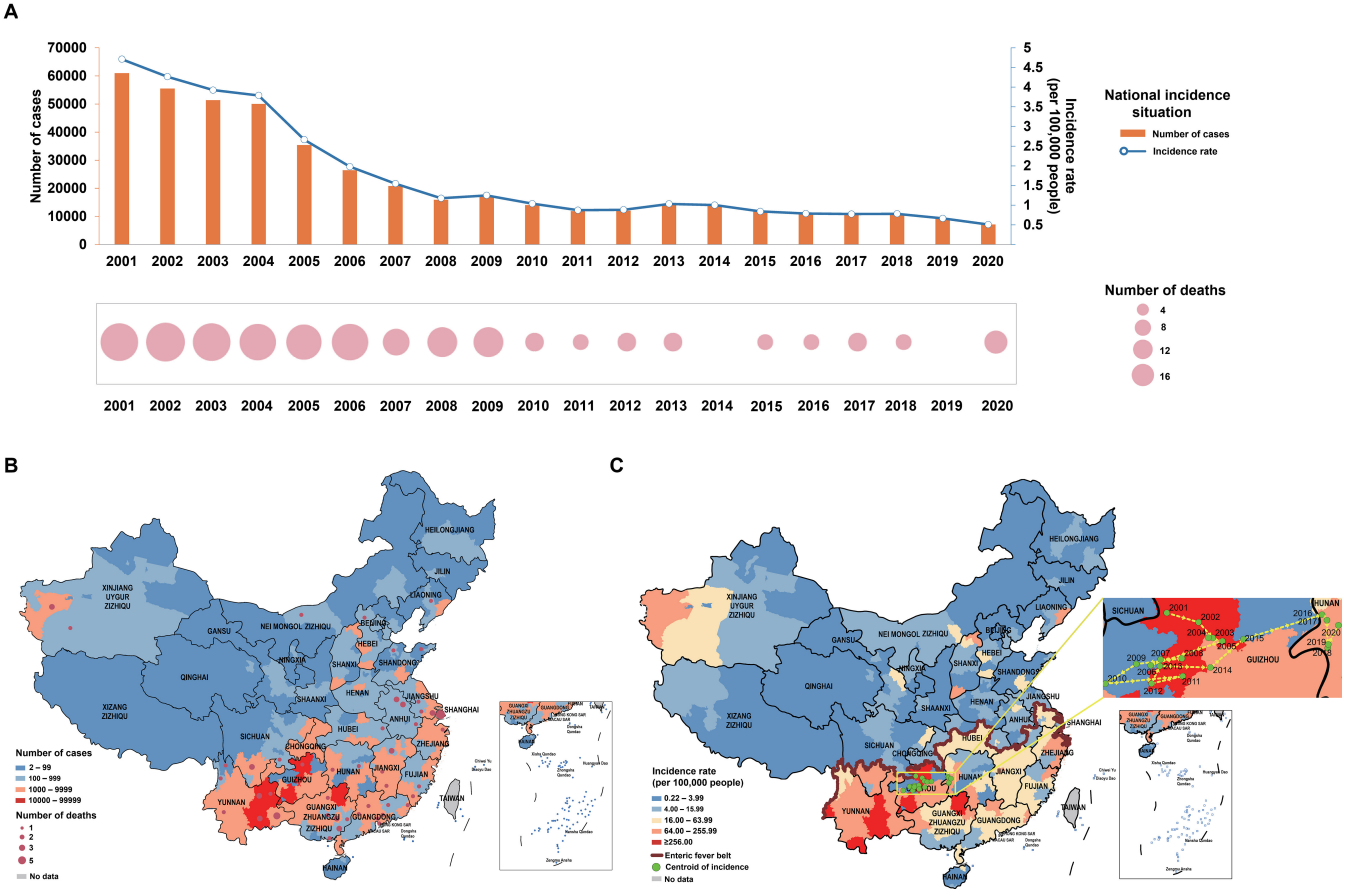
Distribution of enteric fever. **(A)** Number of cases, number of deaths and incidence rate of enteric fever in China from 2001 to 2020; **(B)** number of cases and number of deaths of enteric fever in Chinese cities from 2001 to 2020; **(C)** incidence rate of enteric fever, enteric fever zone, and centroid transfer of incidence rates in Chinese cities from 2001 to 2020.

**Figure 2:**
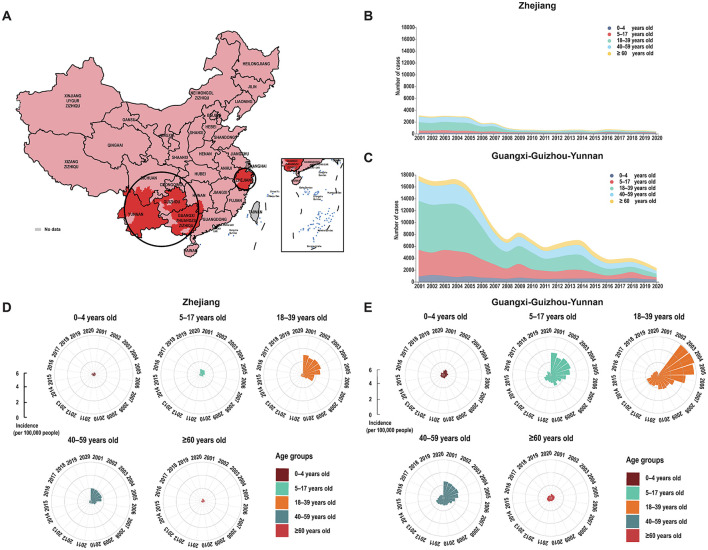
Spatial and age-group patterns of enteric fever in China from 2001 to 2020. **(A)** Spatial clustering of enteric fever in China from 2001 to 2020. **(B)** Number of enteric fever cases by age group in Zhejiang. **(C)** Number of enteric fever cases by age group in Guangxi-Guizhou-Yunnan. **(D)** Incidence of enteric fever by age group in Zhejiang. **(E)** Incidence of enteric fever by age group in Guangxi-Guizhou-Yunnan.

From 2001–2020, 258,087 cases of enteric fever were reported across 293 cities in mainland China, with a significant decline in both cases and incidence rates, particularly between 2004 and 2008. Since 2009, the epidemic has stabilized and deaths have remained relatively low at around four to eight per year (Figure 1A). Approximately 25.86% of cities reported over 1,000 cases, and 35.52% had an incidence rate exceeding 16 per 100,000 people, with notable severity in Guizhou, Yunnan, and Guangxi. High incidence rates were also observed in parts of Zhejiang and Xinjiang, while most cities reported low or no deaths (Figures 1B, C). The distribution of enteric fever in China is uneven, with higher incidence rates primarily in the southern regions. An enteric fever zone, consisting of 131 cities mostly south of the Qin Mountain–Huai River Line, has been identified, contributing to over 90% of the national cumulative cases and incidence rates. From 2001 to 2015, the centroid of incidence was located in western China, but it has shifted eastward since 2016 (Figure 1C). Spatial analysis revealed significant spatial clustering, particularly in the Guangxi–Guizhou–Yunnan and Zhejiang regions, indicating high spatial autocorrelation in these areas (Figure 2A; Supplementary Table 2).

The two clustering areas, Zhejiang and Guangxi–Guizhou–Yunnan, differed in climate and latitude, influencing their incidence rates (Supplementary Table 2). From 2001 to 2005, both clustering areas had stable incidence rates, which then declined, with Guangxi–Guizhou–Yunnan showing a more substantial decrease despite occasional rebounds (Supplementary Figure 1). By 2020, both clustering areas exhibited stabilized case numbers and incidence rates across all age groups, with the disparity between them narrowing over time (Figures 2B–E).

### 3.2 Periodicity and seasonal differences in enteric fever between the two clustering areas

The two clustering areas showed distinct seasonality patterns for enteric fever: in Zhejiang, it peaked between July and August, while in Guangxi–Guizhou–Yunnan, it extended from May to October. Zhejiang exhibited significant 19-month and 61-month periodic changes, with the 19-month cycle being more stable, whereas Guangxi–Guizhou–Yunnan displayed dominant 18-month and 30-month periodic patterns, with the 18-month cycle lasting about seven cycles (Supplementary Figure 2).

### 3.3 Associations of heterogeneous factors with enteric fever incidence rates across the two clustering areas

**Figure 3:**
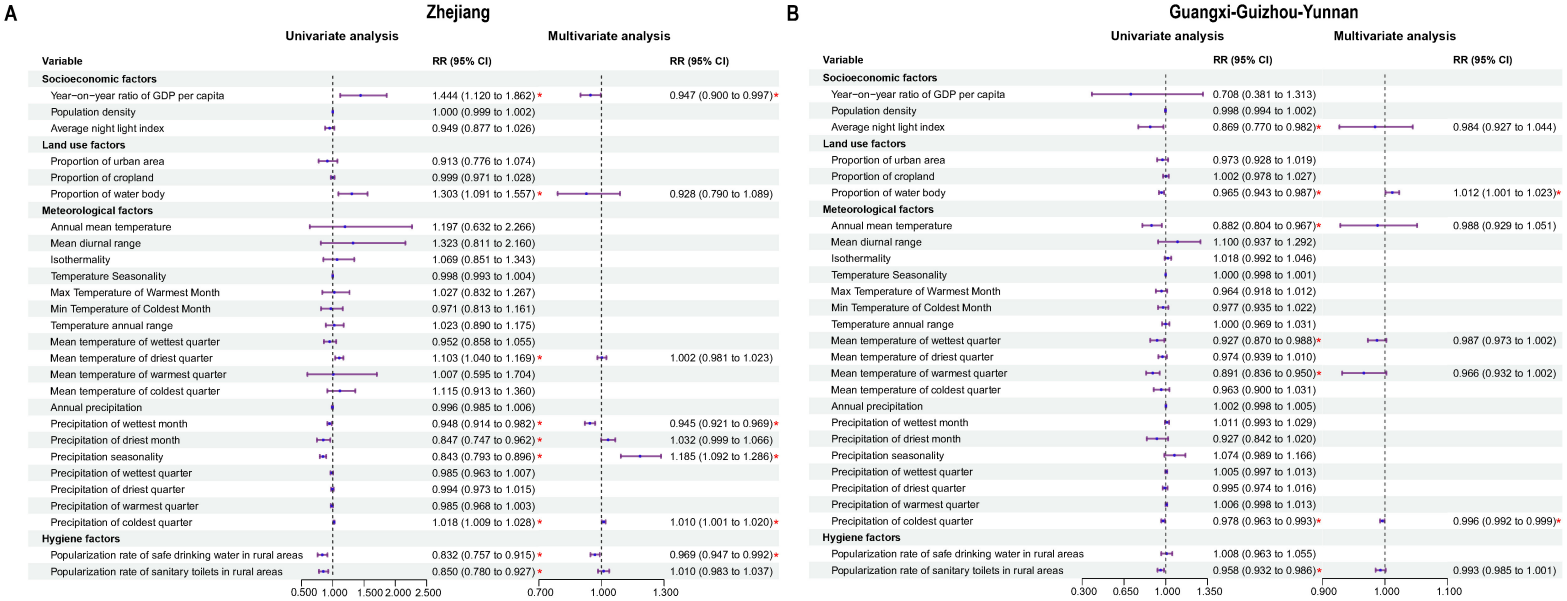
Analysis of influencing factors on enteric fever. **(A)** Potential influencing factors associated with enteric fever in Zhejiang. **(B)** Potential influencing factors associated with enteric fever in Guangxi-Guizhou-Yunnan.

In Zhejiang and Guangxi–Guizhou–Yunnan, different epidemic patterns were observed, with 27 variables evaluated using GEE analysis. In Zhejiang, GDP per capita, precipitation in the wettest month, and the popularization rate of safe drinking water in rural areas were negatively correlated with enteric fever incidence, while precipitation seasonality and precipitation in the coldest quarter were positively correlated; in Guangxi–Guizhou–Yunnan, the precipitation of the coldest quarter was negatively correlated, but the proportion of water bodies showed a positive correlation with enteric fever incidence (Figure 3; Supplementary Tables 3–6).

### 3.4 Lag effects of meteorological factors on enteric fever incidence

**Figure 4:**
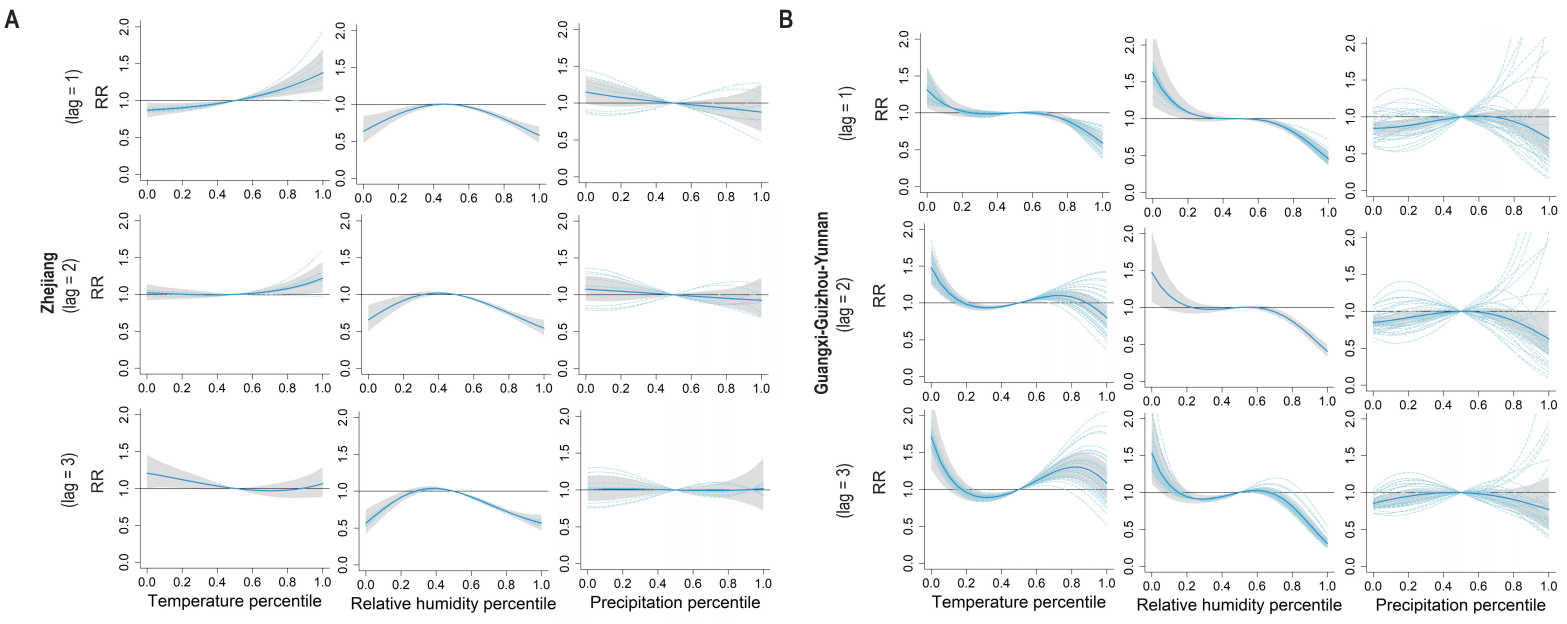
Pooled exposure-response curves for meteorological factors and weekly enteric fever cases with lag times. **(A)** The pooled exposure-response curves between meteorological factors (temperature, relative humidity, percipitation) percentiles and weekly cases of enteric fever with a lag time of 1–3 weeks in Zhejiang. **(B)** The pooled exposure-response curves between meteorological factors (temperature, relative humidity, percipitation) percentiles and weekly cases of enteric fever with a lag time of 1–3 weeks in Guangxi-Guizhou-Yunnan.

In the Zhejiang, a positive effect from the weekly temperature was observed on the incidence of enteric fever, with a 1-week lag effect; when the temperature was below the median level, the effect weakened and even reversed as the lag duration increased (Figure 4A). In the Guangxi–Guizhou–Yunnan, temperature effects were fluctuating, with the risk of enteric fever first decreasing, then increasing, and finally decreasing with increasing temperature over lags of 2–3 weeks (Figure 4B). Elevated relative humidity had negative impacts on enteric fever incidence in both clustering areas across the three lag periods; under low relative humidity conditions, the effect was negative in Zhejiang but positive in Guangxi–Guizhou–Yunnan. No significant lag effect of precipitation on enteric fever incidence was detected in the Zhejiang, whereas in the Guangxi–Guizhou–Yunnan, low precipitation had a weakly positive effect on enteric fever incidence with a lag of 1–3 weeks.

## 4 Discussion

Over the past 20 years in China, a decreasing trend in the prevalence of enteric fever epidemics nationwide has been observed, and socioeconomic and sanitary improvements may play important roles (29). Although these efforts are evident to people, cities in regions of high prevalence still face a continuous threat from enteric fever, which may require stronger targeted measures to contain the epidemic of the disease in these areas. In this study, Guangxi-Guizhou-Yunnan and Zhejiang were identified as two clustering areas for enteric fever, with distinct underlying mechanisms potentially driving the observed epidemiological patterns: The elevated incidence in Guangxi-Guizhou-Yunnan predominantly stems from persistent groundwater contamination due to karst topography, inadequate sanitation infrastructure, and transient interprovincial population aggregation during ethnic festivals (e.g., the March Fair) (10, 13, 19). Conversely, Zhejiang's high incidence correlates strongly with water source pollution from stagnant zones in coastal alluvial plains, sustained transmission chains through cyclical migration of migrant workers (“Spring Festival repatriation + dry season return”), and delayed expansion of health services relative to rapid urbanization demands (11, 12, 15, 18, 30–32). The high-incidence period of enteric fever in Zhejiang clustering area was predominantly concentrated in the summer, resembling a “torrential scouring effect”—intense rainfall during the monsoon season triggers floods that rapidly introduce contaminants from open drainage systems into drinking water supplies, significantly increasing the risk of waterborne transmission. The relationship between the incidence of enteric fever and popularization rate of safe drinking water in rural areas in Zhejiang further supports this observation. In contrast, small-scale outbreaks in the spring were associated with human-to-human transmission caused by social gatherings and dining out during the Chinese New Year (33). In sharp contrast, the high-incidence period in the Guangxi-Guizhou-Yunnan clustering area spans both spring and autumn. This is primarily attributed to the unique “slow filtration” mechanism of karst topography, where porous rock formations allow polluted water to continuously infiltrate underground water systems. Combined with the mild and humid climate during spring and autumn, this facilitates a prolonged cycle of pathogen transmission between groundwater and surface water systems (34). These environmentally driven regional differences highlight the need for tailored prevention strategies: in Zhejiang, efforts should focus on safeguarding drinking water systems during the rainy season, while in the southwestern regions, interventions must address the chronic transmission cycle caused by geological characteristics and the persistent contamination of subterranean water systems. Studies also indicate that the seasonality of enteric fever varies across different latitudes, which aligns with our research findings (35). In-depth periodic analysis revealed that in the Zhejiang clustering area, the 19-month short-term cycle may stem from synergistic effects of El Niño oscillations, peak irrigation demands in rice cultivation systems, and migrant worker mobility patterns, with its statistical prominence underscoring the dominant role of climatic drivers. The 61-month long-term cycle likely reflects dynamic interactions between periodic fluctuations in public health infrastructure efficacy (e.g., phased water supply/sanitation modifications under Five-Year Plans) and Salmonella serovar antigenic drift. In contrast, the Guangxi-Guizhou-Yunnan clustering area exhibited an 18-month short-term cycle potentially linked to biennial intensification of monsoon precipitation and recurrent ethnic festival gatherings, which collectively impose rhythmic environmental-social stresses. The 30-month long-term cycle showed temporal correspondence with fly population dynamics, suggesting possible vector-mediated transmission amplification during demographic resurgence phases of dipteran species. With respect to the discrepancy in enteric fever incidence across age groups, people aged 18–39 years were the main population affected by the enteric fever, which may be related to an increase in their exposure risk attributed to their more frequent social and economic activities (8). A slight increase in the incidence among people aged 5–17 years was observed in the Guangxi–Guizhou–Yunnan from 2016 to 2018, suggesting that the specific risk factors for this age group as well as the necessity of adjustment in vaccine coverage still need to be investigated. The age-specific distribution and spatiotemporal trends of enteric fever exhibit distinct characteristics: individuals aged 18–39 years consistently represent the highest-risk group, with elevated incidence rates strongly associated with frequent socio-economic activities and heightened exposure risks to contaminated water or food sources (8). The decline in incidence observed post-2010 in both studied regions demonstrates the effectiveness of public health interventions, particularly water quality improvement and food safety management. Notably, a marginal increase in incidence among the 5–17 age group was identified in the Guangxi-Guizhou-Yunnan cluster during 2016–2018, suggesting potential unidentified risk factors or insufficient vaccination coverage within this demographic that necessitates targeted investigation. Although lower incidence rates in children and older adult populations may reflect immunization benefits or reduced environmental exposure, persistent vigilance against localized transmission risks remains critical. Future prevention strategies should prioritize high-risk groups (18–39 years) through enhanced vaccination campaigns, health education, and rural water infrastructure upgrades, while maintaining cross-sectoral surveillance to sustain long-term control achievements.

Furthermore, by using GEE models, we assessed and compared the potential influencing factors with enteric fever incidence rates in the two clustering areas. The proportion of water bodies was positively correlated with the incidence rate of enteric fever in the Guangxi–Guizhou–Yunnan. It has been suggested that larger water bodies have more complex drainage networks, causing the accumulation of pollutants along their length and the enrichment of bacteria (25), thus resulting in a greater risk of pathogen exposure and an increased incidence in human populations (36). The GDP per capita in the Zhejiang had a negative impact on the incidence rate of enteric fever, which coincided with another study that reported a positive correlation between the incidence rate of enteric fever and urban poverty, suggesting that economic progress can contribute to the containment of enteric fever transmission (37). Limited sanitation conditions and the use of untreated water or surface water can increase the likelihood of enteric fever transmission, and improved water, sanitation and hygiene (WASH) can provide substantial protection against enteric fever (38, 39). In the Zhejiang, the implementation of the “Thousands of Villages Demonstration, Tens of Thousands of Villages Renovation” and the “Beautiful Countryside” initiatives has increased the use of safe drinking water, likely leading to a reduction in the incidence of enteric fever. These measures have improved infrastructure and the environment, thereby enhancing GDP per capita and the popularization rate of safe drinking water, which in turn have had a negative impact on the incidence of enteric fever (40–42). In the Zhejiang, precipitation during the cold season increased the incidence of enteric fever due to urbanization and topographical features that prolonged water retention time, leading to more severe river pollution (43, 44). Conversely, in the Guangxi–Guizhou–Yunnan, cold season precipitation decreased the incidence of enteric fever because the mountainous and hilly terrain allowed surface runoff to quickly remove pollutants, thus reducing the risk of water source contamination (45). In this study, we found that high temperatures usually positively affected the risk of enteric fever. High temperatures may indirectly promote the occurrence of enteric fever by increasing the reproduction rate of *S*. Typhi, and making people more likely to drink untreated water, while excessive water intake may impair the bactericidal function of gastric acid (34, 46). For precipitation, there appears to be a negligible effect of precipitation on the incidence rates of enteric fever in the two clustering areas, with the exception of conditions characterized by extremely low precipitation, which was aligned with the findings of previous studies (46, 47). There has been considerable controversy regarding whether relative humidity affects the incidence of enteric fever. Our study revealed different effects of low relative humidity on enteric fever incidence in the two clustering areas, with lower relative humidity related to a decreased risk of enteric fever in the Zhejiang and an increased risk in the Guangxi–Guizhou–Yunnan. Some studies have presented conflicting findings concerning the impact of relative humidity on enteric fever (48, 49). Since 2004, China has improved enteric fever monitoring and intervention through the CISDCP and national surveillance sites, although economic development and geographic conditions can impact these efforts (10, 50).

There are several limitations in our study that should be acknowledged. First, the factors influencing the incidence rate of enteric fever may not be fully considered due to the lack of data; for example, changes in behavioral habits related to food safety and public education about enteric fever were not included due to unavailability, which would inevitably introduce a potential bias in the estimation of the relative risk for these factors identified in our study. Second, although our GEE model has advantages in analyzing annual data and potential influencing factors, it has certain limitations; for example, the model may not fully capture short-term temporal changes and complex interactions, which limits the precision of our causal analysis.

## 5 Conclusions

In summary, although the incidence rate of enteric fever has steadily declined over the past 20 years, the rate of decline has significantly slowed since 2009, indicating the onset of a low-level endemic plateau phase. Our findings reveal that the two clustering areas are influenced by distinct risk factors, which exert varying impacts across regions. To address these challenges, we recommend conducting precise, region-specific factor analyses to tailor interventions, particularly in high-incidence regions. Based on the identified risk factors, targeted public health strategies should include: (1) strengthening sanitation infrastructure to reduce fecal-oral transmission (e.g., expanding sewage systems and promoting safe waste disposal); (2) enhancing access to clean water through localized water quality monitoring and household-level treatment solutions. These measures should be integrated into cross-sectoral collaborations involving health authorities, local governments, and community leaders to ensure sustainable impact. By prioritizing context-driven, evidence-based actions, policymakers can accelerate progress toward eradicating enteric fever in high-risk regions.

## Data Availability

The original contributions presented in the study are included in the article/Supplementary material, further inquiries can be directed to the corresponding author.
