# Supplementary material for: Spatiotemporal distribution and influencing factors of enteric fever in China: a cluster analysis based on data from 2001 to 2020
Source: Front Public Health. 2025 Apr 9;13:1550904. doi: 10.3389/fpubh.2025.1550904 (PMC12014557; doi:10.3389/fpubh.2025.1550904)
Supplement: Supplementary file 1 [file Data_Sheet_1.docx]

**Supplementary Materials and Methods**

***SaTScan***

The spatial scan statistical method is currently one of the most commonly used methods for disease cluster analysis. Among these methods, SaTScan proposed by Kulldorff^1^ in 1997 is widely applied and has been used to identify high-risk areas for various diseases^2,3^. The analysis involved calculating the log-likelihood ratio (LLR) of the attributes of spatial units within and outside the dynamic window area under different centers and radii, in order to infer statistical significance and identify clustering centers. A higher LLR value with significant differences indicates a higher probability of the dynamic window area being a cluster area, suggesting a higher likelihood of it being a high-risk area.

***Wavelet transform***

Wavelet transform is currently widely used in periodicity and trend analysis of time series, including discrete wavelet analysis and continuous wavelet analysis. The Morlet wavelet in continuous wavelet analysis was used here. The Morlet wavelet was introduced by Grossmann and Morlet and possesses good time-frequency local properties and high resolution^4^. It is defined as follows:

ψ(t)=π^(-1/4)*exp(jωt)*exp(-t^2/2)

Among them, ψ(t) is the Morlet wavelet function, j is the imaginary unit, ω is the frequency parameter, and t is the time parameter.

***Distributed lag non-linear model***

The Distributed lag non-linear model (DLNM), proposed by Antonio Gasparrini, is commonly used for analyzing data with time lag effects, where the values of the dependent variable are correlated with past values of the independent variable^5^. It is writed as follows:

Log[*E(Y_t_)*]=*cb(X_j_, lag, df)+ns(time, df)+ ns(season, df)+ ∑ns(X_m_, df)*

where *E(Y_t_)* denotes the monthly enteric fever incidence in month t, *cb()* denotes the cross-basic function modeling the impact of *X_j_* (meteorological factor) on enteric fever, *ns()* denotes the cubic spline function, time denotes the days to control for the long-term, season denotes the seasonal trend of enteric fever, and *X_m_* denotes meteorological factors that are not in the cross basis function.

***Restricted Maximum Likelihood and multivariate meta-analysis***

Restricted Maximum Likelihood (REML) is a method for variance component analysis, primarily used in mixed effects models, to estimate the contribution of each random effect to the variance of the dependent variable. By computing the variance components, it is possible to identify the key focus objects for reducing variance and correcting for the downward bias of variance components. Estimation is performed by maximizing a special case of the expression λ_REML_ given by Jennrich and Schluchter^6^:

λ_REML_=$-\frac{1}{2}\sum_{i=1}^{n} \log\left| S_{i}+\Sigma\right|-\frac{1}{2}\log\left| \sum_{i=1}^{n} \left( S_{i}+\Sigma\right)^{-1} \right|- \frac{1}{2}\sum_{i=1}^{n} r_{i}^{T}\left( S_{i}+\Sigma\right)^{-1}\gamma_{i}$

λ_REML_ is the log-likelihood of the residual, $S_{i}$ are the covariance matrix of vector of effects, $\gamma_{i}$ are the residuals, $\Sigma$ is the between-study covariance matrix.

Compared to univariate meta-analysis, multivariate meta-analysis has the advantage of being able to describe the associations between effect. In longitudinal data meta-analysis, when the effects of interest exist at multiple time points, it is important to consider the correlation between the effects when modeling the trend of the effects over time. Studies have shown that ignoring the correlation between the time points of each effect in univariate meta-analysis can lead to overestimation or underestimation of the effects^7^. Additionally, multivariate meta-analysis can estimate the summary effect, which is not possible with univariate meta-analysis. The expression is given as :

$\hat{u}=(\sum_{i=1}^{n} ({S_{i}+{\hat{\Sigma})}^{-1})}^{-1}(\sum_{i=1}^{n} (S_{i}+{\hat{\Sigma})}^{-1}Y_{i}$)

$\hat{u}$ is the pooled estimate, $Y_{i}$ is the vector of effects, $S_{i}$ are the within-study covariance matrices, ∑ is between-study covariance matrices.

***Generalized estimating equation***

For each individual with only one observed value, generalized linear model (GLM) can be applied. However, for longitudinal datasets with repeated observations, it is necessary to consider the correlation between the given individual values, in which case the GLM is no longer applicable. In 1986, Liang and Zeger proposed Generalized estimating equation (GEE) ^8^. GEE is an extension of GLM, with the main difference being that GLM focuses on the specific individual's effects and assumes that the distribution of the dependent variable follows an exponential family distribution, while GEE focuses on estimating the average effects of the population without making any distributional assumptions. GEE estimates the model parameters by transforming commonly used distributions such as binomial, Poisson, and Gamma distributions through a link function, thus obtaining the corresponding statistical models. GEE effectively addresses the issue of correlation among individual observed values in longitudinal data, making full use of the data information and reducing the loss of information. The formula for the GEE model is expressed as follows:

$g(\mu_{ij}$)=$\beta_{0}+\beta_{1}X_{ij1}+\beta_{2}X_{ij2}+\ldots+\beta_{p}X_{ijp}$

The logarithmic value of the incidence count in the i-th year and j-th city is denoted as $\mu_{ij}$, where g() represents the link function and $\beta$=($\beta_{1}\ldots\beta_{p}$) represents the parameter vector to be estimated in the model.

**Supplementary Reference**

1. Kulldorff M. A spatial scan statistic. Commun Stat Theory Methods. 2007;26(6):1481-1496. doi: 10.1080/03610929708831995.

2. Coleman M, Coleman M, Mabuza AM, et al. Using the SaTScan method to detect local malaria clusters for guiding malaria control programmes. Malar J. 2009;8:68. Published 2009 Apr 17. doi:10.1186/1475-2875-8-68.

3. Adjemian J, Olivier KN, Seitz AE, et al. Spatial clusters of nontuberculous mycobacterial lung disease in the United States. Am J Respir Crit Care Med. 2012;186(6):553-558. doi:10.1164/rccm.201205-0913OC.

4. Grossmann A, Morlet J. Decomposition of functions into wavelets of constant shape, and related transforms. In: Mathematics + Physics. 1985:135-165. doi:10.1142/9789814415125_0004.

5. Gasparrini A, Armstrong B, Kenward MG. Distributed lag non-linear models. Stat Med. 2010;29(21):2224-2234. doi:10.1002/sim.3940.

6. Schluchter J M D .Unbalanced Repeated-Measures Models with Structured Covariance Matrices. Biometrics. 1986; 42(4):805-820. doi:10.2307/2530695.

7. Hedges LV, Tipton E, Johnson MC. Robust variance estimation in meta-regression with dependent effect size estimates. Res Synth Methods. 2010;1(1):39-65. doi: 10.1002/jrsm.5.

8. Liang K Y , Zeger S L. Longitudinal data analysis using generalized linear models.Biometrika. 1986; 73(1):13-22. doi:10.2307/2336267.

**eTable 1. Units note of influencing factors.**

| **Variable** | **Unit note** |
| --- | --- |
| Year-on-year ratio of GDP per capita | - |
| Population density | People per 10,000 square kilometers |
| Average night light index | - |
| Proportion of urban area | /10 |
| Proportion of cropland | /10 |
| Proportion of water body | /10 |
| Annual mean temperature | ℃ |
| Mean diurnal range | ℃ |
| Isothermality | - |
| Temperature Seasonality | - |
| Max Temperature of Warmest Month | ℃ |
| Min Temperature of Coldest Month | ℃ |
| Temperature annual range | ℃ |
| Mean temperature of wettest quarter | ℃ |
| Mean temperature of driest quarter | ℃ |
| Mean temperature of warmest quarter | ℃ |
| Mean temperature of coldest quarter | ℃ |
| Annual precipitation | 10mm |
| Precipitation of wettest month | 10mm |
| Precipitation of driest month | 10mm |
| Precipitation seasonality | 10mm |
| Precipitation of wettest quarter | 10mm |
| Precipitation of driest quarter | 10mm |
| Precipitation of warmest quarter | 10mm |
| Precipitation of coldest quarter | 10mm |
| Popularization rate of safe drinking water in rural areas | % |
| Popularization rate of sanitary toilets in rural areas | % |

**eTable 2. Spatial Aggregation of Enteric Fever in China from 2001 to 2020.**

| **Clustering areas** | **Radius (km)** | ***LLR*** | ***P*-value** | **Risk population** |
| --- | --- | --- | --- | --- |
| Guangxi-Guizhou-Yunnan clustering area | 861 | 9126.89 | <0.001 | 115,696,935 |
| Zhejiang clustering area | 224 | 1225.35 | <0.001 | 46,975,945 |

LLR: Log-likelihood ratio.

**eTable 3. Univariate GEE Model Analysis of the Zhengjiang Clustering Area.**

| **Subgroup** | **Coefficients** | ***P*** | ***RR*** | **95% *CI*** |
| --- | --- | --- | --- | --- |
| **Socioeconomic factors** |  |  |  |  |
| Year-on-year ratio of GDP per capita | 0.367 | 0.005 | 1.444 | (1.120,1.862) |
| Population density | 0.000 | 0.718 | 1.000 | (0.999,1.002) |
| Average night light index | -0.053 | 0.184 | 0.949 | (0.877,1.026) |
| **Land use factors** |  |  |  |  |
| Proportion of urban area | -0.091 | 0.271 | 0.913 | (0.776,1.074) |
| Proportion of cropland | -0.001 | 0.945 | 0.999 | (0.971,1.028) |
| Proportion of water body | 0.265 | 0.003 | 1.303 | (1.091,1.557) |
| **Meteorological factors** |  |  |  |  |
| Annual mean temperature | 0.180 | 0.581 | 1.197 | (0.632,2.266) |
| Mean diurnal range | 0.280 | 0.263 | 1.323 | (0.811,2.160) |
| Isothermality | 0.067 | 0.567 | 1.069 | (0.851,1.343) |
| Temperature Seasonality | -0.002 | 0.575 | 0.998 | (0.993,1.004) |
| Max Temperature of Warmest Month | 0.026 | 0.807 | 1.027 | (0.832,1.267) |
| Min Temperature of Coldest Month | -0.029 | 0.748 | 0.971 | (0.813,1.161) |
| Temperature annual range | 0.022 | 0.754 | 1.023 | (0.890,1.175) |
| Mean temperature of wettest quarter | -0.050 | 0.348 | 0.952 | (0.858,1.055) |
| Mean temperature of driest quarter | 0.098 | 0.001 | 1.103 | (1.040,1.169) |
| Mean temperature of warmest quarter | 0.007 | 0.979 | 1.007 | (0.595,1.704) |
| Mean temperature of coldest quarter | 0.108 | 0.286 | 1.115 | (0.913,1.360) |
| Annual precipitation | -0.004 | 0.406 | 0.996 | (0.985,1.006) |
| Precipitation of wettest month | -0.054 | 0.003 | 0.948 | (0.914,0.982) |
| Precipitation of driest month | -0.166 | 0.010 | 0.847 | (0.747,0.962) |
| Precipitation seasonality | -0.171 | 0.000 | 0.843 | (0.793,0.896) |
| Precipitation of wettest quarter | -0.015 | 0.174 | 0.985 | (0.963,1.007) |
| Precipitation of driest quarter | -0.006 | 0.559 | 0.994 | (0.973,1.015) |
| Precipitation of warmest quarter | -0.015 | 0.097 | 0.985 | (0.968,1.003) |
| Precipitation of coldest quarter | 0.018 | 0.000 | 1.018 | (1.009,1.028) |
| **Hygiene factors** |  |  |  |  |
| Popularization rate of safe drinking water in rural areas | -0.184 | 0.000 | 0.832 | (0.757,0.915) |
| Popularization rate of sanitary toilets in rural areas | -0.162 | 0.000 | 0.850 | (0.780,0.927) |

**eTable 4. Multivariate GEE Model Analysis of the Zhengjiang Clustering Area.**

| **Subgroup** | **Coefficients** | ***P*** | ***RR*** | **95% *CI*** |
| --- | --- | --- | --- | --- |
| **Socioeconomic factors** |  |  |  |  |
| Year-on-year ratio of GDP per capita | -0.055 | 0.037 | 0.947 | (0.900,0.997) |
| Population density |  |  |  |  |
| Average night light index |  |  |  |  |
| **Land use factors** |  |  |  |  |
| Proportion of urban area |  |  |  |  |
| Proportion of cropland |  |  |  |  |
| Proportion of water body | -0.075 | 0.358 | 0.928 | (0.790,1.089) |
| **Meteorological factors** |  |  |  |  |
| Annual mean temperature |  |  |  |  |
| Mean diurnal range |  |  |  |  |
| Isothermality |  |  |  |  |
| Temperature Seasonality |  |  |  |  |
| Max Temperature of Warmest Month |  |  |  |  |
| Min Temperature of Coldest Month |  |  |  |  |
| Temperature annual range |  |  |  |  |
| Mean temperature of wettest quarter |  |  |  |  |
| Mean temperature of driest quarter | 0.002 | 0.872 | 1.002 | (0.981,1.023) |
| Mean temperature of warmest quarter |  |  |  |  |
| Mean temperature of coldest quarter |  |  |  |  |
| Annual precipitation |  |  |  |  |
| Precipitation of wettest month | -0.057 | 0.000 | 0.945 | (0.921,0.969) |
| Precipitation of driest month | 0.031 | 0.058 | 1.032 | (0.999,1.066) |
| Precipitation seasonality | 0.170 | 0.000 | 1.185 | (1.092,1.286) |
| Precipitation of wettest quarter |  |  |  |  |
| Precipitation of driest quarter |  |  |  |  |
| Precipitation of warmest quarter |  |  |  |  |
| Precipitation of coldest quarter | 0.010 | 0.031 | 1.010 | (1.001,1.020) |
| **Hygiene factors** |  |  |  |  |
| Popularization rate of safe drinking water in rural areas | -0.031 | 0.007 | 0.969 | (0.947,0.992) |
| Popularization rate of sanitary toilets in rural areas | 0.010 | 0.472 | 1.010 | (0.983,1.037) |

**eTable 5. Univariate GEE Model Analysis of the Guangxi-Guizhou-Yunnan Clustering Area.**

| **Subgroup** | **Coefficients** | ***P*** | ***RR*** | **95% *CI*** |
| --- | --- | --- | --- | --- |
| **Socioeconomic factors** |  |  |  |  |
| Year-on-year ratio of GDP per capita | -0.346 | 0.273 | 0.708 | (0.381,1.313) |
| Population density | -0.002 | 0.253 | 0.998 | (0.994,1.002) |
| Average night light index | -0.140 | 0.024 | 0.869 | (0.770,0.982) |
| **Land use factors** |  |  |  |  |
| Proportion of urban area | -0.028 | 0.248 | 0.973 | (0.928,1.019) |
| Proportion of cropland | 0.002 | 0.881 | 1.002 | (0.978,1.027) |
| Proportion of water body | -0.036 | 0.002 | 0.965 | (0.943,0.987) |
| **Meteorological factors** |  |  |  |  |
| Annual mean temperature | -0.126 | 0.008 | 0.882 | (0.804,0.967) |
| Mean diurnal range | 0.095 | 0.245 | 1.100 | (0.937,1.292) |
| Isothermality | 0.018 | 0.175 | 1.018 | (0.992,1.046) |
| Temperature Seasonality | 0.000 | 0.798 | 1.000 | (0.998,1.001) |
| Max Temperature of Warmest Month | -0.037 | 0.136 | 0.964 | (0.918,1.012) |
| Min Temperature of Coldest Month | -0.023 | 0.312 | 0.977 | (0.935,1.022) |
| Temperature annual range | 0.000 | 0.979 | 1.000 | (0.969,1.031) |
| Mean temperature of wettest quarter | -0.076 | 0.020 | 0.927 | (0.870,0.988) |
| Mean temperature of driest quarter | -0.027 | 0.149 | 0.974 | (0.939,1.010) |
| Mean temperature of warmest quarter | -0.115 | 0.000 | 0.891 | (0.836,0.950) |
| Mean temperature of coldest quarter | -0.037 | 0.282 | 0.963 | (0.900,1.031) |
| Annual precipitation | 0.002 | 0.331 | 1.002 | (0.998,1.005) |
| Precipitation of wettest month | 0.011 | 0.248 | 1.011 | (0.993,1.029) |
| Precipitation of driest month | -0.076 | 0.120 | 0.927 | (0.842,1.020) |
| Precipitation seasonality | 0.071 | 0.089 | 1.074 | (0.989,1.166) |
| Precipitation of wettest quarter | 0.005 | 0.206 | 1.005 | (0.997,1.013) |
| Precipitation of driest quarter | -0.005 | 0.633 | 0.995 | (0.974,1.016) |
| Precipitation of warmest quarter | 0.006 | 0.127 | 1.006 | (0.998,1.013) |
| Precipitation of coldest quarter | -0.022 | 0.005 | 0.978 | (0.963,0.993) |
| **Hygiene factors** |  |  |  |  |
| Popularization rate of safe drinking water in rural areas | 0.008 | 0.736 | 1.008 | (0.963,1.055) |
| Popularization rate of sanitary toilets in rural areas | -0.043 | 0.003 | 0.958 | (0.932,0.986) |

**eTable 6. Multivariate GEE Model Analysis of the Guangxi-Guizhou-Yunnan Clustering Area.**

| **Subgroup** | **Coefficients** | ***P*** | ***RR*** | **95% *CI*** |
| --- | --- | --- | --- | --- |
| **Socioeconomic factors** |  |  |  |  |
| Year-on-year ratio of GDP per capita |  |  |  |  |
| Population density |  |  |  |  |
| Average night light index | -0.016 | 0.596 | 0.984 | (0.927,1.044) |
| **Land use factors** |  |  |  |  |
| Proportion of urban area |  |  |  |  |
| Proportion of cropland |  |  |  |  |
| Proportion of water body | 0.012 | 0.033 | 1.012 | (1.001,1.023) |
| **Meteorological factors** |  |  |  |  |
| Annual mean temperature | -0.012 | 0.706 | 0.988 | (0.929,1.051) |
| Mean diurnal range |  |  |  |  |
| Isothermality |  |  |  |  |
| Temperature Seasonality |  |  |  |  |
| Max Temperature of Warmest Month |  |  |  |  |
| Min Temperature of Coldest Month |  |  |  |  |
| Temperature annual range |  |  |  |  |
| Mean temperature of wettest quarter | -0.013 | 0.096 | 0.987 | (0.973,1.002) |
| Mean temperature of driest quarter |  |  |  |  |
| Mean temperature of warmest quarter | -0.034 | 0.067 | 0.966 | (0.932,1.002) |
| Mean temperature of coldest quarter |  |  |  |  |
| Annual precipitation |  |  |  |  |
| Precipitation of wettest month |  |  |  |  |
| Precipitation of driest month |  |  |  |  |
| Precipitation seasonality |  |  |  |  |
| Precipitation of wettest quarter |  |  |  |  |
| Precipitation of driest quarter |  |  |  |  |
| Precipitation of warmest quarter |  |  |  |  |
| Precipitation of coldest quarter | -0.004 | 0.022 | 0.996 | (0.992,0.999) |
| **Hygiene factors** |  |  |  |  |
| Popularization rate of safe drinking water in rural areas |  |  |  |  |
| Popularization rate of sanitary toilets in rural areas | -0.007 | 0.087 | 0.993 | (0.985,1.001) |


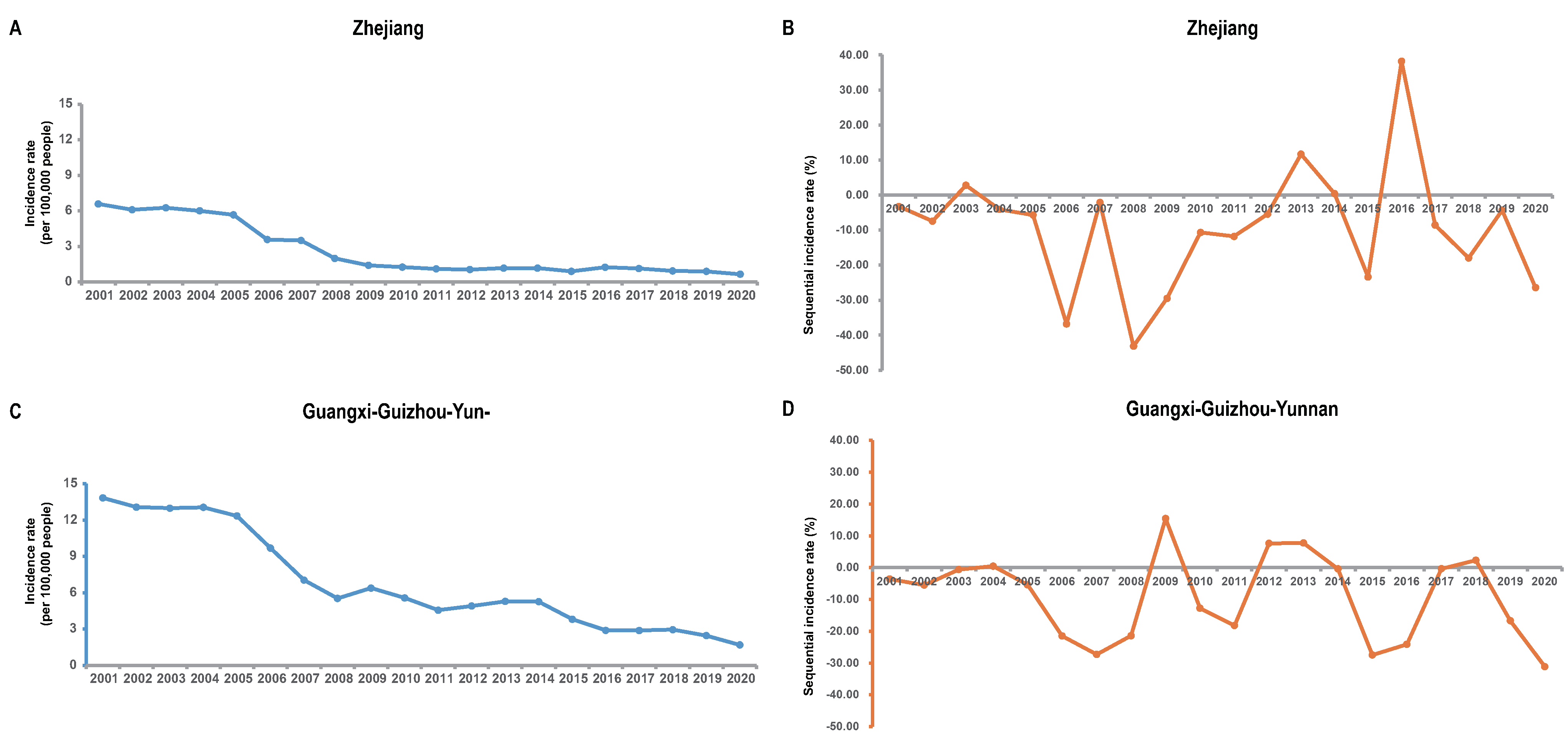


**eFigure 1. Incidence rate and sequential incidence rate of enteric fever in Zhejiang and Guangxi-Guizhou-Yunnan clustering areas from 2001 to 2020.** (A) Incidence rate of enteric fever in Zhejiang clustering area from 2001 to 2020; (B) Sequential incidence rate of enteric fever in ​​Zhejiang clustering area from 2001 to 2020; (C) Incidence rate of enteric fever in ​​Zhejiang clustering area from 2001 to 2020; (D) Sequential incidence rate of enteric fev in Guangxi-Guizhou-Yunnan clustering area from 2001 to 2020.


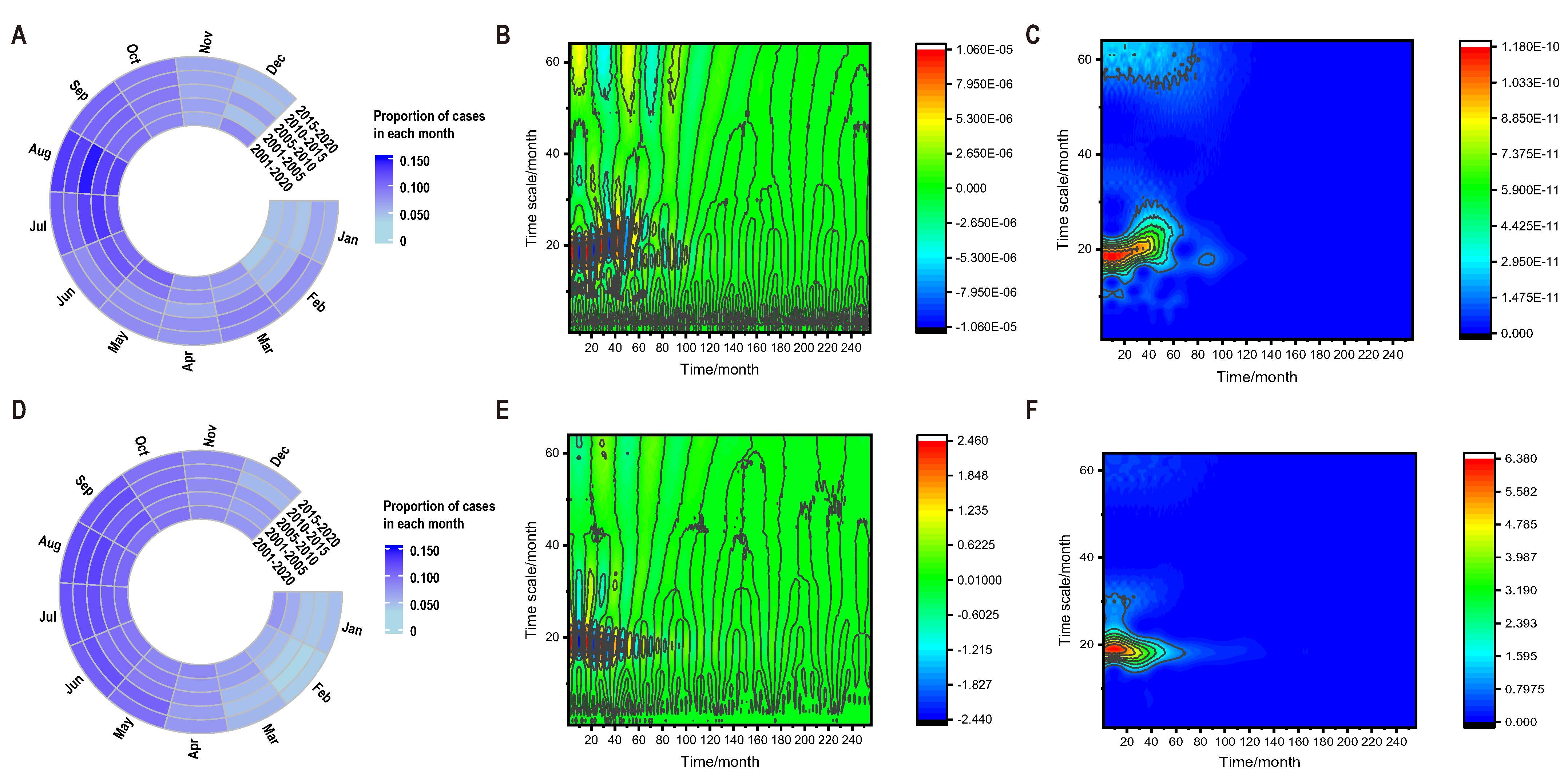


**eFigure 2. Periodic and seasonal characteristics of enteric fever epidemic in Zhejiang and Guangxi-Guizhou-Yunnan clustering areas from 2001 to 2020.** (A) Monthly composition ratio of enteric fever cases in Zhejiang clustering area from 2001 to 2020; (B) Real part of the complex wavelet coefficients of enteric fever monthly incidence in ​​Zhejiang clustering area from 2001 to 2020; (C) Modulus of the complex wavelet coefficients of enteric fever incidence in ​​Zhejiang clustering area from 2001 to 2020; (D) Monthly composition ratio of enteric fever cases in Guangxi-Guizhou-Yunnan clustering area from 2001 to 2020; (E) Real part of the complex wavelet coefficients of enteric fever monthly incidence in Guangxi-Guizhou-Yunnan clustering area from 2001 to 2020; (F) Modulus of the complex wavelet coefficients of enteric fever incidence in Guangxi-Guizhou-Yunnan clustering area from 2001 to 2020.
